# Tracing contact and migration in pre-Bantu Southern Africa through lexical borrowing

**DOI:** 10.1017/ehs.2025.10014

**Published:** 2025-07-23

**Authors:** Anne-Maria Fehn, Bonny E. Sands, Admire Phiri, Maitseo Bolaane, Gaseitsiwe Masunga, Ezequiel Fabiano, Jorge Rocha

**Affiliations:** 1 CIBIO, Centro de Investigação em Biodiversidade e Recursos Genéticos, InBIO Laboratório Associado, Campus de Vairão, Universidade do Porto, Porto, Portugal; 2 Biopolis Program in Genomics, Biodiversity and Land Planning, CIBIO, Campus de Vairão, Vairão, Portugal; 3 Department of English, Northern Arizona University, Flagstaff, AZ, USA; 4 Department of Linguistics, University of the Free State, Bloemfontein, South Africa; 5 San Research Centre, University of Botswana, Gaborone, Botswana; 6 Okavango Research Institute, University of Botswana, Maun, Botswana; 7 University of Namibia, Windhoek, Namibia; 8 Departamento de Biologia, Faculdade de Ciências, Universidade do Porto, Porto, Portugal

**Keywords:** Southern African prehistory, Southern African Khoisan, evolutionary linguistics, quantitative linguistics, lexical borrowing, language contact

## Abstract

Lexical borrowing may provide valuable clues about the sociohistorical context of language contact. Here we explore patterns of vocabulary transfer between languages from three families (Kx’a, Tuu, Khoe-Kwadi) comprising the linguistic unit commonly referred to as Southern African Khoisan. In our data set, 20% of 1,706 roots are shared between at least two families. By applying a carefully chosen set of linguistic and extralinguistic criteria, we were able to trace the origin of 71% of shared roots, with the remaining 29% constituting good candidates for ancient contact or shared common ancestry of the forager families Kx’a and Tuu. More than half of the shared roots for which an origin could be determined trace back to Khoe-Kwadi and were borrowed into languages of other families within two major confluence zones with different sociohistorical profiles: (i) the Central Kalahari characterized by egalitarian interaction between languages of all three families and (ii) the southern and south-western Kalahari Basin fringes showing unilateral transfer from Khoe-Kwadi-speaking herders into resident forager groups. The findings of this study complement genetic and archaeological research on southern Africa and testify to the value of linguistics in the multidisciplinary inference of contact and migration scenarios.

## Graphical abstract

**Figure:**
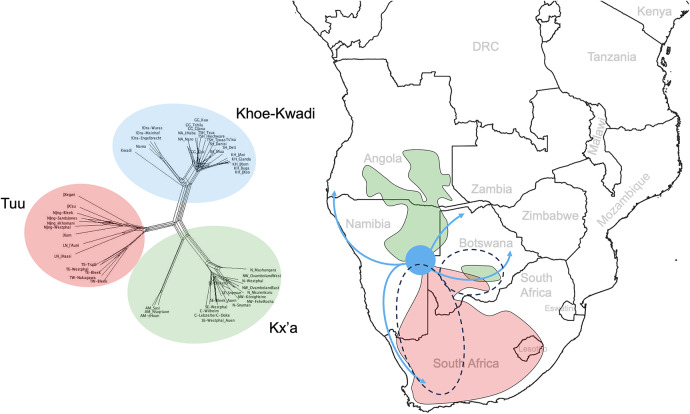

## Social media summary

Lexical borrowing between click languages of three distinct families provides new insights into the pre-history of southern Africa.

## 1. Introduction

Multidisciplinary research findings combining evidence from linguistics, archaeology and genetics support the existence of three major population layers in pre-colonial southern Africa (Fehn et al., 2022): (i) an ancient foraging layer presently represented by speakers of Kx’a and Tuu languages; (ii) a Late Stone Age migration of sheep herders from eastern Africa associated with languages of the Khoe-Kwadi family; and (iii) the agriculture-related dispersal of Bantu-speaking Iron Age farmers from West Central Africa.

The pre-Bantu languages of the Kx’a, Tuu and Khoe-Kwadi families all have unusually large phoneme inventories and share cross-linguistically rare click sounds which are also attested in the Tanzanian languages Hadza and Sandawe. Based on this feature and other superficial commonalities observed during the mass comparison of fragmented data sources, Greenberg (1963) grouped all non-Bantu, non-Cushitic click languages of southern and eastern Africa into a macro-family he dubbed ‘Khoisan’. Although the concept of a ‘Khoisan’ family remains widely used among non-linguists, scholars actively working on the languages in question have failed to back up the genealogical validity of this claim by means of the comparative method or morphological reconstruction (Güldemann, 2008a, 2014a; Sands, 1998; Westphal, 1971). Instead, bottom-up reconstruction involving newly collected data from a variety of languages has led to the establishment and widespread acceptance of three southern African clades (Kx’a, Tuu, Khoe-Kwadi) and two eastern African isolates (Hadza, Sandawe) (Fehn & Rocha, 2023; Güldemann, 2005, 2014a; Heine & Honken, 2010; Vossen, 1997). Although the possibility of a deeper-level relationship between Khoe-Kwadi and Sandawe on the one hand and Kx’a and Tuu on the other has been entertained, no conclusive reconstruction has been attempted for either link (Güldemann, 2014a).

Despite the present inability to show a genealogical connection, it is widely acknowledged that languages of the Kx’a, Tuu and Khoe-Kwadi families share many typological features, including similar sound inventories and syllabic constraints (Güldemann, 2008b; Güldemann & Fehn, 2017; Nakagawa et al, 2023). They are therefore thought to have a history of long-term interaction in a linguistic contact area roughly delimited by the Kalahari Basin, which is here referred to by the term ‘Southern African Khoisan’ (SAK) (Güldemann, 1998, 2014a). Within this area, Kx’a and Tuu are closer to one another than to Khoe-Kwadi.

Previous studies on the role of contact in forming the Kalahari Basin linguistic area mostly concentrated on the transfer of typological features from resident Kx’a and Tuu foragers to incoming Khoe-Kwadi-speaking herders (Güldemann, 2006; Güldemann & Fehn, 2017). Güldemann and Fehn (2017) suggest a gradient of adherence to the SAK typological profile, with Khoe-Kwadi languages spoken in and around the Central Kalahari displaying more influence from Kx’a and Tuu than their relatives which remained at the fringes of the contact area. However, advances in the documentation of individual Khoe-Kwadi languages have shown that some features previously ascribed to relatively recent Kx’a and Tuu influence are actually widespread across the family, indicating the SAK profile may have extended well into eastern Africa, or was obtained at the Proto-Khoe-Kwadi stage, immediately after reaching southern Africa (Fehn & Phiri, 2022; Fehn & Rocha, 2023).

Despite their possibly contact-induced adherence to the Kalahari Basin typological profile, it has been implicitly assumed that Khoe-Kwadi speakers, due to their pastoral subsistence, became dominant in the region before the arrival of Bantu-speaking farmers. However, there is little evidence to support this model. Genetically, Kx’a and Tuu speakers had considerable influence on the genetic profile of modern Khoe-Kwadi speakers, all of whom display varying degrees of ancestry derived from southern African forager groups while retaining only small fragments of their original eastern African genetic heritage (Oliveira et al, 2023; Pickrell et al, 2014). Furthermore, only speakers of the Khoekhoe and Kwadi branches of Khoe-Kwadi have been associated with pastoralism in historical times (Barnard, 1992; Fehn & Rocha, 2023).

The situation within the Kalahari Khoe – the family’s biggest and most diverse branch – is more complex: although the raising of small-stock has been observed among speakers of Kalahari Khoe in the Central Kalahari since the 1960s (Ikeya, 1993; Russell, 2017; Tanaka, 1969), the focus on goats suggests a Bantu origin of herding practices, rather than a retention of an older, sheep-centric, pattern. Furthermore, all Kalahari Khoe speakers, irrespective of their current access to livestock, share a foraging lifestyle and worldview with their Kx’a and Tuu-speaking neighbours (Barnard, 1992). Whereas the present situation testifies to the relative simplicity in which hunter-gatherers may pick up and integrate livestock into their traditional lifeways (Russell, 2017), the striking differences between the subsistence patterns of the Khoekhoe on the one hand and the Kalahari Khoe on the other have led to intense speculation on whether modern Kalahari Khoe speakers are former herders who lost their livestock, or simply Kx’a- and Tuu-speaking hunter-gatherers who changed their language (Fehn et al, 2022; Güldemann, 2008b). In a unifying attempt, it has further been suggested that the ancestors of the Khoe-Kwadi were hunter-herders who led a life combining foraging and livestock-keeping practices, effectively allowing their descendants to switch between both subsistence practices in response to environmental and societal triggers (Fehn et al, 2022; Sadr, 2008).

As lexical borrowing – that is, the horizontal transfer of vocabulary from one language to another – is often associated with cultural innovations or elevated prestige of one language over another (Thomason & Kaufman, 1988), the vocabulary domain seems especially prone to provide insights into social relations between Kx’a, Tuu and Khoe-Kwadi speakers in the pre-Bantu population landscape of southern Africa. However, no large-scale quantitative assessment of lexical borrowing across the entire SAK area has been attempted, even though the cross-family sharing of vocabulary has long been noted and significantly contributed to Greenberg’s (1963) original ‘Khoisan’ proposal (Gerlach, 2016; Güldemann & Loughnane, 2012; Sands, 1998, 2001; Traill & Nakagawa, 2000). More generally, the historical implications of lexical borrowing across large-scale multilingual data sets have been neglected in favour of assessing the borrowability of semantic fields and meanings (e.g. Kelih, 2024; Tadmor et al, 2010; van Hout & Muysken, 1994), as well as on the automatic detection of borrowings in data sets used for genealogical language classification (e.g., (List & Forkel, 2022; Nelson-Sathi et al, 2011). Still, lexical borrowings are more than just an obstacle to uncover the true relationships between languages; they contain part of a language’s history, thereby granting us a glimpse into the history of its speakers and the way they interacted with others.

**Figure 1.**
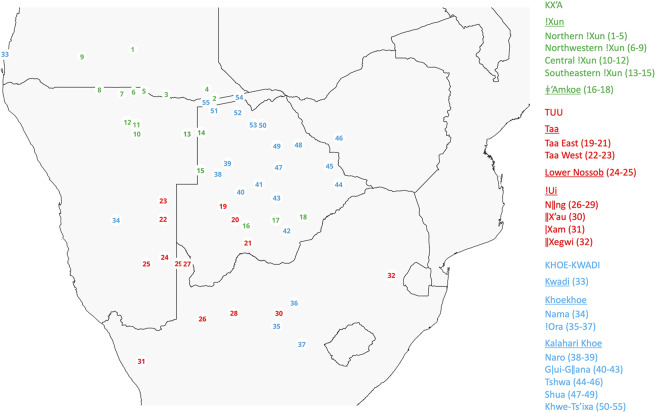
Map showing the approximate geographic origin of each data set used in the present study. Individual data sets are identified by language family and number as referenced in Supplementary Table S1. The legend groups doculects into language families and widely accepted subgroups (cf. Fehn & Rocha, 2023; Güldemann, 2014a; Heine & Honken, 2010; Heine & König, 2015; Vossen, 1997).

In order to uncover the history behind the complex network of lexical borrowing in the Kalahari Basin, we here apply a bottom–top approach which notably differs from mass comparison based on superficial similarity in that the detection of borrowings is combined with the careful historical-comparative reconstruction of lexical roots for each previously established genealogical unit (Kx’a, Tuu, Khoe-Kwadi), taking into account known and newly detected patterns of sound correspondence. To this end, we devised a set of diverse criteria for establishing the direction of transfer: these include language-internal criteria linked to reconstructability, attestation and phonological complexity which allow us to detect violation of established rules of sound change within each family, as well as additional criteria like the presence of alternative roots, historical parsimony and geography which may help to decide on more complex cases for which phonological criteria remain inconclusive. Rather than focusing on the small number of Kx’a, Tuu and Khoe-Kwadi languages for which dictionaries are available, we base our analysis on a unique data set consisting of 57 doculects including published, archival and newly collected data from both vital and extinct languages which belong to all three families and cover a wide geographic area spanning Angola, Namibia, Botswana, Zimbabwe and South Africa, today known as Southern African Development Community (SADC; Fig. 1, Supplementary Table S1). Using 228 meanings encompassing words from the Swadesh 200 list of basic vocabulary and additional meanings widely attested across southern African languages (Supplementary Table S2), we identify a total of 1,706 lexical roots (Supplementary Table S3), 20% of which are shared between languages from at least two families (Supplementary Table S4). We were able to identify 71% of shared roots as borrowings, that is, roots for which an origin within a given family, as well as a distinct direction of transfer (e.g. from Kx’a to Khoe-Kwadi), could be determined. Of those, 41% originated within the Khoe-Kwadi family, whereas only 20% and 10% originated in Kx’a and Tuu respectively. Roots that originated in Khoe-Kwadi are most prominent in languages of the Tuu family, whose speakers were in contact with Khoekhoe-speaking herders, suggesting elevated prestige of food producers among resident forager groups. The data further yielded 29% of shared roots for which no origin could be determined. We do not treat them as borrowings but consider them unresolved cases that may be indicative of ancient contact, or a distant common ancestor shared by Kx’a and Tuu, in line with previous proposals (Collins & Honken, 2016; Güldemann, 2014a). We note that our findings on borrowing also hold if a deep genealogical relationship between all three families (Kx’a, Tuu, Khoe-Kwadi) is assumed, given that such a relationship would significantly pre-date the historical movements under consideration, and go much beyond the presently established sound correspondences which form the basis of our analysis.

The findings of this study complement genetic and archaeological research on southern Africa as a contact zone and testify to the value of linguistics in the multidisciplinary inference of contact and migration scenarios.

## 2. Material and methods

### 2.1. Assembly of the data set

This study is based on a list of 228 meanings initially assembled for 63 doculects from the Kx’a, Tuu and Khoe-Kwadi families, from study locations in Angola, Namibia, Botswana, Zimbabwe and South Africa (Fig. 1). The wordlist consists of 132 meanings from the Swadesh 200 wordlist of basic vocabulary (Swadesh, 1952) and 96 additional meanings for which we could obtain good data coverage, particularly for under-attested and extinct languages of the !Ui (Tuu) cluster. Swadesh 200 meanings that were either (a) function words (grammatical items like pronouns or adverbs), (b) for which the data coverage was insufficient or (c) which have no generic expression in the data at hand (like ‘leg’ or ‘burn’) were removed.

The database combines newly collected data from Kx’a (Northern !Xun: Mashangara, Nkurenkuru; Northwestern !Xun: Mupa) and Khoe-Kwadi (Shua: Deti, Shua, Danisi; Khwe: ǁAni, Buga, Gǀanda, ǁXoo, Ts’ixa) with archival and published sources. The name, linguistic affiliation, subsistence pattern, sampling location and bibliographical source for each doculect is provided in Supplementary Table S1.

This study was developed within the TwinLab network of collaborations linking CIBIO/InBIO to ISCED (Instituto Superior de Ciências de Educação)–Huíla, Angola, to the University of Namibia, and to the Okavango Research Institute and the San Research Center of the University of Botswana. All newly collected data were gathered with official research permits issued by the Governments of Namibia and Botswana, and with the support and permission of the Provincial Governments of the Namibe and Kunene provinces (Angola).

### 2.2. Coding of the linguistic data

The linguistic data were coded as follows: for every meaning, we identified all roots attested in the data set, based on previous works and general knowledge of sound correspondences attested in the SAK languages (see Supplementary Text S1 and Supplementary Table S5 for a complete list of all historical-comparative works consulted). We then coded each language for the presence (1), absence (0), or lack of information (?) for a given root. Synonyms were allowed, that is, a language was permitted to have more than one root for a given meaning. However, a root can appear only once in the data set, even if it is attested with different meanings within the universe of our 228-meaning list. These cases of polysemy are always marked in the data set and filed under the cross-linguistically most widespread meaning of each root. Bantu borrowings and complex expressions (e.g. ‘chest bone’ for ‘rib’) were removed. To achieve a better data coverage in the !Ui group of Tuu, the three doculects for ǂKhomani (Doke, 1936; Köhler, n.d..; Maingard, 1937) and the five doculects for ǁXegwi (Bleek, 1956; Köhler, n.d..; Lanham & Hallowes, 1956; Westphal, 1953-1971; Ziervogel, 1955) were lumped into a single doculect, respectively. The division between individual sources is maintained in the lexical database (Supplementary Table S2). The final data set which all analyses are based on consists of 1,706 roots, based on 228 meanings, which were coded for a total of 57 doculects from the Kx’a, Tuu and Khoe-Kwadi families (Supplementary Table S3).

### 2.3. NeighborNet analysis

**Figure 2.**
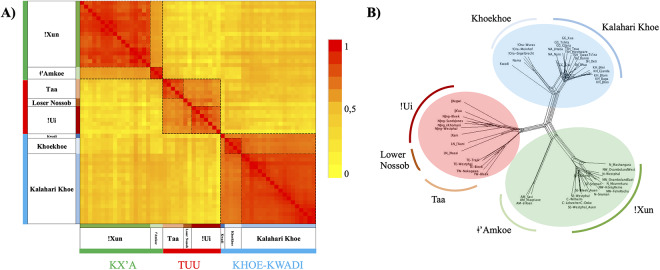
Language relationships as assessed with the GeneContent distance (Gray et al., 2010); both the heatmap based on linguistic similarity (a) and the NeighborNet (b) clearly identify the three families Kx’a (green), Tuu (red) and Khoe-Kwadi (blue), as well as their major subgroups. Lexical sharing between the three families is evident as reticulation in the centre of the network.

To create a network graph of our data set (Fig. 2B, Supplementary Figure S1), we used the NeighborNet algorithm implemented in the software SplitsTree 4.17.2 (Huson & Bryant, 2006), applying GeneContent distance and filtering weights at 0.005 as recommended by Gray et al. (2010). We further created a corresponding heatmap based on a similarity matrix 1 – GeneContent distance, which was visualized in MS Excel (Fig. 2A).

### 2.4. Bayesian phylogenetic analysis

We performed a Bayesian phylogenetic analysis for each language family (Kx’a, Tuu, Khoe-Kwadi) by importing the ascertained binary data in the software Beast 2.5 (Bouckaert et al, 2019). In order to obtain a basic phylogeny and posterior support values for individual language clusters, we opted for the Continuous Markov Chain Model (CTMC) included in the Babel package (https://github.com/rbouckaert/Babel) under a Yule model, following a strict clock. We acknowledge possible caveats of this simplified model choice (Hoffmann et al., 2021), but as we do not use priors with the aim to date our phylogeny, we consider our analysis satisfactory for the purpose of this work. We ran each analysis for 20 million generations, during which we could obtain adequate values for effective sample sizes (ESS) of all parameters. Consensus trees were subsequently visualized in FigTree v.1.4.4 (Rambaut, 2018).

### 2.5. Establishing the source for shared roots

To determine the genealogical origin for each root, we defined a set of criteria adapted to the SAK linguistic area that take into account previous guidelines for the identification of borrowings (e.g. Güldemann & Loughnane, 2012; Haspelmath, 2009). We identified five primary criteria (reconstructability, scope of attestation, phonology and morphological analysability) as well as four secondary criteria (presence of lexical alternatives, geography, culture and historical parsimony). To determine the origin and direction of transfer for a given root, we usually employed more than one criterion. Details and examples for each criterion are provided in Supplementary Text S1.

### 2.6. Analyses of lexical sharing

**Figure 3.**
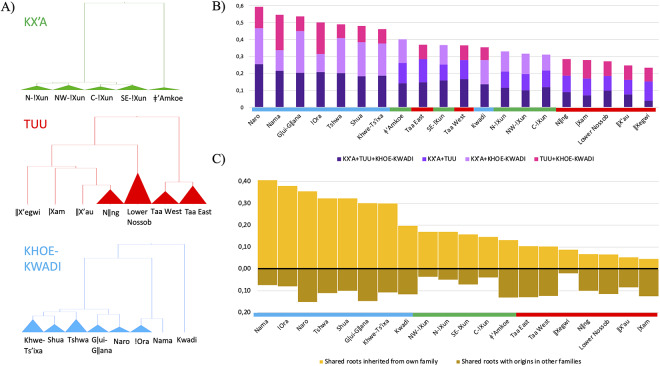
(a) Bayesian phylogenetic trees used to identify major language clusters within each family. (b) Proportion of roots with different sharing patterns in each language cluster. (c) Percentage of roots that were transferred from one’s own family to other families (top bars) or that originated in other families; that is, loanwords (bottom bars). All proportions refer to fractions of total roots per language cluster. The horizontal bars below the bar plots indicate the language family: Kx’a (green), Tuu (red) and Khoe-Kwadi (blue).

Analyses of lexical sharing are based on roots shared between doculects from at least two families (Supplementary Table S4), comprising four types of sharing patterns: KX’A + TUU + KHOE-KWADI; KX’A + KHOE-KWADI; TUU + KHOE-KWADI; KX’A + TUU (Fig. 3B). Percentages of sharing were obtained for each doculect, and subsequently averaged across doculects forming a language cluster. Language clusters were established according to posterior probabilities obtained through a Bayesian phylogenetic analysis as shown in Supplementary Figures S2–4.

## 3. Results

### 3.1. Distance-based and Bayesian phylogenetic analyses

In a distance-based analysis, our data set reproduces the expected division into distinct families while clearly showing lexical overlap between all three of them (Fig. 2). In both the heatmap (Fig. 2A) and the NeighborNet analysis (Fig. 2B), the Kx’a, Tuu and Khoe-Kwadi families are distinguished, along with all known subgroups within each family: Kx’a is divided into two distinct branches, !Xun and ǂ’Amkoe (Heine & Honken, 2010); Tuu has two clearly defined branches, Taa and !Ui, as well as the Lower Nossob language cluster with unclear affiliation within the family (Güldemann, 2005; Starostin, 2021, 2022); Khoe-Kwadi consists of the outlier language Kwadi and the Khoe branch divided into the Khoekhoe and Kalahari Khoe subgroups (Fehn & Rocha, 2023; Güldemann, 2004; Vossen, 1997). The NeighborNet analysis further shows box-like structures in the centre of the network (Fig. 2B), indicating reticulation and therefore lexical sharing between languages of all three families. Reticulation is also visible within the !Xun branch of Kx’a and the Kalahari Khoe subgroup of Khoe-Kwadi, respectively, possibly pointing to family-internal contact between related languages spoken in close geographic proximity. The clear division into major families and subgroups also holds if singletons are removed from the data set, indicating that the differentiation within SAK is driven by broader sharing patterns and not by micro-variation within single doculects (Supplementary Figure S1).

To assess how the doculects included in our study align with previously identified language clusters within each family, we also performed a Bayesian phylogenetic analysis for Kx’a, Tuu and Khoe-Kwadi (Fig. 3A, Supplementary Figures S2–4). In accordance with other studies, the consensus tree for Kx’a shows a deep split between !Xun and ǂ’Amkoe (Heine & Honken, 2010), as well as a relatively shallow differentiation within each branch (Supplementary Figure S2). Our findings further replicate previous research on Kx’a subclassification (Heine & König, 2015; Sands, 2010; Snyman, 1997) in rendering support for a single ǂ’Amkoe cluster, made up of three doculects, as well as for four !Xun clusters, namely Northern, North-western, Central and South-eastern !Xun, consisting of at least three doculects each. The Tuu phylogeny clearly delimits a Taa branch divided into a western and an eastern cluster, and an !Ui branch, consisting of the Nǁng cluster including Nǀuu and ǂKhomani, as well as the isolated doculects ǁX’au, ǀXam and ǁXegwi (Güldemann, 2014a; Supplementary Figure S3). The position of Lower Nossob (here grouped with !Ui) is problematic (see also Starostin, 2022): Lower Nossob shares grammatical features with Taa and has been included in a genealogical unit termed Taa-Lower Nossob (Güldemann, 2014b). The lexical affinities between Lower Nossob and !Ui may therefore be a secondary feature, related to family-internal language contact in the region of what is now the Kgalagadi Transfrontier National Park.

In line with a previous Bayesian phylogenetic analysis (Fehn et al., 2022), our tree for Khoe-Kwadi clearly replicates the major division between the isolated Kwadi branch and the Khoe languages, which are further subdivided into Khoekhoe and Kalahari Khoe (Supplementary Figure S4). Within Khoekhoe, the Nama language can be distinguished from the !Ora cluster represented by three closely related doculects. The Kalahari Khoe subgroup consists of five language clusters: Gǀui-Gǁana, Naro, Khwe-Ts’ixa, Shua and Tshwa.

Overall, we observe a close resemblance between the topologies inferred by qualitative linguistic studies and the results of our phylogenetic analyses, despite the obvious presence of contact-induced reticulation in the data set (cf. Greenhill et al., 2009).

## 4. Quantitative assessment of roots shared between at least two families

In the analyses of lexical sharing in SAK that follow, the languages and language clusters identified in the Bayesian study are used as units of comparison (Fig. 3A).

The affiliation of individual doculects to distinct language clusters is provided in Supplementary Table S1. In a first step, we identified all roots that appear in languages of more than one family. These amount to 20% (340/1,706) of the full data set. As much as 27% of shared roots were found in all three families, 21% in Kx’a and Tuu, 23% in Tuu and Khoe-Kwadi and 29% in Kx’a and Khoe-Kwadi. The distribution of shared roots across languages from different families shows that the highest percentage of shared roots is attested in languages of the Khoe branch of Khoe-Kwadi, reaching up to 60% in Naro (Fig. 3B).

We then determined the source family for each root, following the criteria outlined in Supplementary Text S1. We found that 41% of shared roots had originated within the Khoe-Kwadi family, whereas only 20% and 10% had originated within Kx’a and Tuu, respectively. Our criteria did not allow us to identify a definite origin for 29% of shared roots, which will be discussed further below. When ranked by the percentage of shared roots that originated in one’s own family, the Khoe-Kwadi languages rank first, displaying sharing rates between 41% (Nama) and 20% (Kwadi); languages of the Kx’a family (17–13%) rank next, followed by the Tuu family (11–5%) (Fig. 3C).

Most languages in our data set share more roots through transfer from their own to other families than through borrowing (Fig. 3C). The highest percentages of borrowed vocabulary are displayed by languages of all three families spoken in the Central Kalahari, namely Naro, Gǀui-Gǁana (Khoe-Kwadi, 15% each), ǂ’Amkoe (Kx’a, 13%) and Taa East (Tuu, 13%). Relatively high amounts of borrowed vocabulary are also found in languages of the Tuu family spoken along the western Kalahari Basin fringe, namely Taa West (13%), ǀXam (13%) and Lower Nossob (12%). The lowest percentages of borrowings are found in languages of the !Xun branch of Kx’a spoken in the northwest (7–4%), as well as in the easternmost Tuu language ǁXegwi (2%).

**Figure 4.**
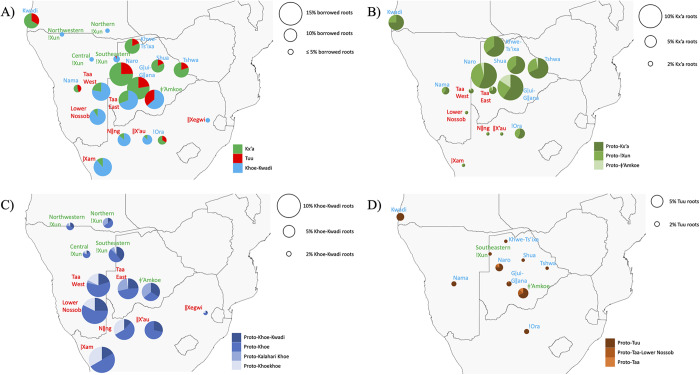
Borrowing profiles of Kx’a, Tuu and Khoe-Kwadi languages. The size of the pie chart corresponds to the percentage of borrowed vocabulary, as indicated for each figure. (a) shows the composition of the borrowed vocabulary in each language; (b) shows Kx’a borrowings in languages from the Tuu and Khoe-Kwadi families, broken down to the highest resolution level that could be obtained for each root (Proto-Kx’a, Proto-!Xun, Proto-ǂ’Amkoe); (c) shows Khoe-Kwadi borrowings in languages from the Kx’a and Tuu families, broken down to the highest resolution level that could be obtained for each root (Proto-Khoe-Kwadi, Proto-Khoe, Proto-Kalahari Khoe, Proto-Khoekhoe); (d) shows Tuu borrowings in languages from the Kx’a and Khoe-Kwadi families, broken down to the highest resolution level that could be obtained for each root (Proto-Tuu, Proto-Taa-Lower Nossob, Proto-Taa).

When borrowings in individual languages are broken down by family of origin (Fig. 4A), distinct, family-internal patterns emerge.

In Kx’a (Supplementary Figure S5A), languages of the !Xun branch spoken on the north-western Kalahari Basin fringe display only few borrowings, which are almost exclusively associated with the Khoe-Kwadi family. In contrast, the ǂ’Amkoe branch located in the Central Kalahari of Botswana has considerably more loanwords that derive from both Khoe-Kwadi and Tuu, reflecting the linguistic diversity of its neighbours.

In the Tuu family (Supplementary Figure S6A), the Taa branch of the Central and Western Kalahari derives about three-quarters of its borrowed vocabulary from Khoe-Kwadi, and about one-quarter from Kx’a. Further south, languages of the !Ui branch display mostly loanwords of Khoe-Kwadi origin, which are considerably more frequent in the western part of South Africa than in areas further east.

In the Khoe-Kwadi family (Supplementary Figure S7A), loanword frequencies are highest in Naro and Gǀui-Gǁana spoken in the Central Kalahari; intermediate in Kwadi, Khwe-Ts’ixa, Shua and Tshwa spoken along the northern Kalahari Basin fringe; and lowest in the Khoekhoe varieties spoken by Nama and !Ora herders in Namibia and South Africa. Although loanwords in Khoe-Kwadi languages predominantly trace back to Kx’a, Tuu contributions are equally present in all three main branches (Kwadi, Khoekhoe, Kalahari Khoe), in line with previous suggestions of both Kx’a and Tuu influence on the ancestral language Proto-Khoe-Kwadi (Fehn & Rocha, 2023).

In order to assess the most recent contact layer for individual languages from different geographic regions, we divided the identified loanwords by their resolution level within the family of origin (Fig. 4B–D), assuming that roots which can be reconstructed to lower-level subgroups would provide additional information on the affiliation of the donor.

Applying this approach, we found that roots with a Kx’a origin (Fig. 4B) were predominantly spread by languages of the !Xun subgroup. Borrowings from !Xun are common in languages of the Khoe-Kwadi family (Supplementary Figure S7B), including those spoken in areas far to the east (Shua, Tshwa) and south (!Ora) of regions where !Xun languages are presently attested. This observation either suggests a different distribution of !Xun in the past, or ancient contact affecting now extinct proto-languages (Proto-Khoe-Kwadi, Proto-Khoe), rather than their living descendants. The latter scenario would support a movement of Kalahari Khoe and Khoekhoe speakers from west to east, at odds with earlier proposals (Barnard, 1992; Elphick, 1985) but in line with the archaeological record (Lander & Russell, 2018) and genetic patterns (Fehn et al., 2022; Oliveira et al, 2023). Kx’a-borrowings tracing back to ǂ’Amkoe are mostly found in Gǀui-Gǁana, in support of a localized borrowing network in the Central Kalahari (Gerlach, 2016; Güldemann & Loughnane, 2012; Traill & Nakagawa, 2000).

In accordance with the observation that the majority of shared roots (41%) originated within Khoe-Kwadi, we find evidence for a widespread influence of this family on other southern African languages. The distribution of Khoe-Kwadi borrowings (Fig. 4C) shows influence of both the Khoekhoe and Kalahari Khoe subgroups on Kx’a (Supplementary Figure S5B) and Tuu languages (Supplementary Figure S6B) from different geographic regions. The spread of Khoekhoe-associated borrowings shows a striking overlap with the known distribution of Khoekhoe-speaking herders, namely along the Orange River (Nǁng), on the west coast of South Africa towards the Cape (ǀXam) and across Namibia up to the border with Angola (Lower Nossob, Taa West, !Xun). The intensive borrowing of Khoe-Kwadi lexis among Tuu languages in the southwest reflects the long presence of Khoe-Kwadi herders in this area, as attested by the archaeological evidence for the early spread of livestock along the Atlantic coastline (Lander & Russell, 2018). Further north, only little Khoe-Kwadi influence can be detected in the !Xun subgroup of Kx’a, likely reflecting the relatively recent northward spread of Nama speakers (Barnard, 1992). Contrasting with the widespread influence of Khoekhoe in the west, Kalahari Khoe influence is restricted to Taa East, ǂ’Amkoe and South-eastern !Xun, spoken in and around the Central Kalahari, as well as to Northern !Xun, which has a history of contact with speakers of the Khwe-Ts’ixa language cluster.

Compared to Khoe-Kwadi and Kx’a, the contributions of Tuu vocabulary to languages of other families are minor (Fig. 4D). Notable exceptions are contributions to ǂ’Amkoe (Supplementary Figure S5C) and Naro (Supplementary Figure S7C), both of which most likely borrowed via their geographically close neighbour Taa within the contact network of the Central Kalahari.

## 5. Distribution of roots with unidentified origins

**Figure 5.**
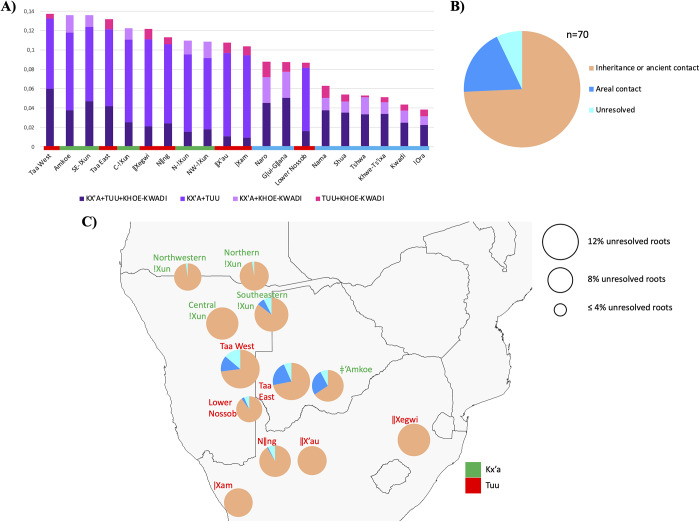
(a) Percentages of roots with unidentified origin in each language cluster, according to their sharing patterns. (b) Pie chart decomposing roots with unidentified origin shared between Kx’a and Tuu, and between Kx’a, Tuu and Khoe-Kwadi (*n*=70), according to whether they are shared: (i) between geographically unconnected units (inheritance or ancient contact); (ii) areally between neighbours (areal contact) or (iii) in an unresolvable setting (unresolved); (c) map showing the geographic distribution of the categories shown in (B) across our Kx’a and Tuu data set. The size of the pie chart corresponds to the percentage of shared roots with unidentified origins in each language cluster, according to the legend.

As indicated above, our data set contains ∼29% (100/340) of shared roots for which no origin could be determined by applying the criteria outlined in Supplementary Text S1; we therefore treated them as unresolved cases of root-sharing, rather than borrowings with a clear origin and direction of transfer. Across the data set, shared roots with unidentified origin are more frequent in languages of the Kx’a and Tuu families than in the Khoe-Kwadi family (Fig. 5A). Moreover, 70% (70/100) of roots with unidentified origins involve sharing between Kx’a and Tuu (Kx’a + Tuu + Khoe-Kwadi, Kx’a + Tuu). Of these, 19% (13/70) can still be explained by contact, as they are shared between neighbouring languages spoken in close geographic proximity, especially within the micro-contact zone of the Central Kalahari (Gerlach, 2016; Güldemann & Loughnane, 2012; Traill & Nakagawa, 2000). Additional 74% (52/70) appear in geographically distant languages or are shared between Proto-Kx’a and Proto-Tuu, thereby suggesting distant common ancestry of the two families, or ancient contact in areas that are presently not occupied by Kx’a and Tuu speakers (Fig. 5B,C). Finally, 7% (5/70) constitute outlier cases that appear in isolated languages across the entire area, but cannot be convincingly ascribed to Kx’a, Tuu or Khoe-Kwadi.

## 6. Discussion

The prehistory of southern Africa is characterized by migration and contact between resident forager populations and incoming food producers successively introducing herding, agriculture and metal working (Fehn et al., 2022). Although multiple studies have shed light on the migration routes and ethnolinguistic impact of Bantu-speaking farmers from West Central Africa (Fortes-Lima et al, 2024; Grollemund et al, 2015; Rocha & Fehn, 2016), contact between diverse forager populations as well as the migratory pathways and social influence of early herders remain poorly understood (Fehn et al, 2022; Lander & Russell, 2018; Sadr, 2008; Smith, 2022). Here, we have explored sharing and exchange of vocabulary between languages of the Kx’a, Tuu and Khoe-Kwadi families in order to learn more about contact events and the sociohistorical conditions that characterized pre-Bantu southern Africa.

Kx’a, Tuu and Khoe-Kwadi share click consonants and a particular phonotactic profile (Güldemann, 2008b; Güldemann & Fehn, 2017; Nakagawa et al., 2023). Hence, detecting the origin of a lexical root attested widely across the area poses a challenge to standard methodology from contact linguistics, which relies heavily on the formal differences between borrowed and inherited vocabulary (compare, e.g., Haspelmath & Tadmor, 2009). We therefore opted to use methods from historical-comparative linguistics that place heavy emphasis on the detection of regular versus irregular phonological correspondences, along with a set of linguistic and extralinguistic criteria, to quantify instances of lexical borrowing between the Kx’a, Tuu and Khoe-Kwadi families (Supplementary Text S1).

In our data set comprising linguistic data from 57 doculects, we were able to identify 20% of roots which are attested in more than one language family (Fig. 3B). As this study relied heavily on core vocabulary and therefore did not include semantic fields known for their high borrowability (e.g., terms for local fauna, landscape features), our result constitutes a conservative estimate which may increase if entire dictionaries, rather than just wordlists, are considered. Furthermore, the considerable percentage (29%) of shared roots for which no origin could be assigned include many roots for which an origin in either Kx’a or Tuu is equally plausible (Fig. 5). The close link between Kx’a and Tuu is in line with evidence from archaeology and genetics which hints at a long-term continuity in the population landscape of southern Africa before food production (Sadr, 2015; Schlebusch et al., 2017, 2020). Although it is widely agreed that Kx’a and Tuu form a stronger typological unit with one another than with the Khoe-Kwadi family (Güldemann, 2008b; Güldemann & Fehn, 2017), a deep genealogical relationship or long-term contact in an ancient linguistic area both seem equally plausible explanations for the currently observed similarities (Collins & Honken, 2016; Güldemann, 2014a).

Following an unknown period of exclusive interaction between Kx’a and Tuu languages, the appearance of sheep dating back to around 2000 BP points to an early introduction of herding from eastern Africa, which has since been associated with languages of the Khoe-Kwadi family (Güldemann, 2008b; Lander & Russell, 2018; Smith, 2022). There can be little doubt that the introduction of small-stock pastoralism must have confronted local foragers with a previously unknown way of life. Yet, it has proven difficult to measure the social impact of the Khoe-Kwadi on the basis of archaeological or genetic evidence. Although criteria to distinguish between forager and herder sites have been proposed (Lander & Russell, 2020; Sadr, 2008, 2015), an overturn in material culture following the arrival of the livestock is still disputed. Furthermore, all modern Khoe-Kwadi speakers broadly share the genetic profiles of their Kx’a, Tuu and Bantu-speaking neighbours, making it difficult to assess their demographic impact on the region (Fehn et al., 2022; Montinaro et al., 2017; Oliveira et al., 2023; Pickrell et al., 2012). In addition, the only Khoe-Kwadi speakers for whom a pronounced pastoralist identity and way of life has been documented in historical times are the Khoekhoe of South Africa and Namibia. In contrast, Kalahari Khoe speakers are historically foragers who adopted small-scale pastoralism only recently (Barnard, 1992; Ikeya, 1993; Russell, 2017; Tanaka, 1969), while the Kwadi of Angola do not preserve an ancient herder profile but mirror the pastoral culture of their Bantu-speaking Kuvale and Himba neighbours (Fehn et al., 2022).

Whereas 41% of all shared roots have a likely Khoe-Kwadi origin and therefore testify to the considerable impact the newcomers had on the Kalahari Basin linguistic area (Fig. 3C), the family-internal division between dominant herding and egalitarian forager populations is also reflected in our results, which suggest differentiated contact scenarios, according to distinct geographic regions.

**Figure 6.**
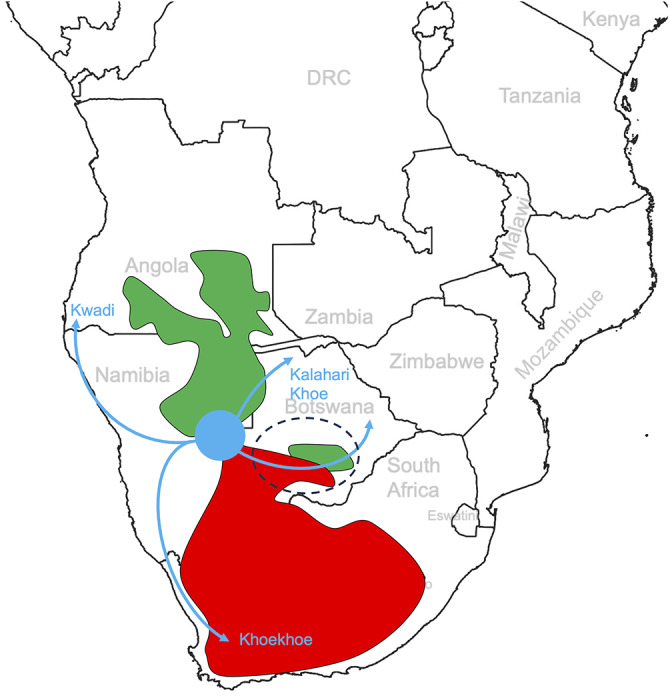
Hypothetical migration routes of Kwadi, Kalahari Khoe and Khoekhoe speakers, out of a core on the western Kalahari Basin fringe where Proto-Khoe-Kwadi and Proto-Khoe emerged (in blue). Historically attested Kx’a- and Tuu-speaking areas are indicated in green and red, respectively. The micro-contact zone of the Central Kalahari where languages of all three families exchange loanwords is encircled by a dashed line.

Along the western and southern Kalahari Basin fringes, Kx’a and Tuu languages receive Khoe-Kwadi roots overwhelmingly from the Khoekhoe subgroup of Khoe, which has been linked to large-scale cattle pastoralism well into historical times (Fig. 4C, Supplementary Figures S5B, S6B). In contrast Khoekhoe only displays residual amounts of Kx’a- and Tuu-derived vocabulary (Figs 4B,D, 6C), indicating that borrowing in this area was a unilateral process.

In the Central Kalahari, on the other hand, languages spoken by Kalahari Khoe foragers (Gǀui-Gǁana, Naro) transfer lexical items to Taa East (Tuu) and the Kx’a languages ǂ’Amkoe and South-eastern !Xun (Fig. 4D, Supplementary Figures S5B, S6B), while also adopting many loanwords from their neighbours (Fig. 4B,D, Supplementary Figure S7). Therefore, the Central Kalahari forms a micro-contact area characterized by egalitarian patterns of exchange between languages from all three families, probably due to shared livelihoods, intermarriage and multilingualism (Barnard, 1992; Gerlach, 2016; Güldemann & Loughnane, 2012; Traill & Nakagawa, 2000).

As Kx’a and Tuu languages spoken in regions outside the influence zone of the Khoekhoe or the Central Kalahari (i.e. North-western, Northern and Central !Xun, ǁXegwi) only display residual amounts of Khoe-Kwadi loanwords (Fig. 4C, Supplementary Figures S5B, S6B), a large-scale impact of the family beyond areas with historically attested contact can likely be excluded.

Beyond characterizing different contact areas, Kx’a and Tuu borrowings into Khoe-Kwadi provide valuable insights into the internal migrations of the Khoe-Kwadi language family inside the Kalahari Basin area, following their initial arrival from eastern Africa. As Khoe-Kwadi languages spoken in regions where no Kx’a and Tuu languages are currently attested (i.e. Kwadi, Khwe-Ts’ixa, Shua, Tshwa) still have 7–9% of Kx’a and 2–4% of Tuu loanwords (Fig. 4B,D, Supplementary Figure S7), we can assume that part of the Kx’a and Tuu lexicon found currently in languages of the Khoe-Kwadi family traces back to historical borrowing events affecting the proto-languages Proto-Khoe, Proto-Kalahari Khoe and Proto-Khoekhoe, which took place in geographic locations where no contact is presently attested. In this frame, we hypothesize that Proto-Khoe-Kwadi must have been spoken in an area which, at the time, was populated by Kx’a and Tuu speakers (see also Fehn & Rocha, 2023; Oliveira et al., 2023), whereas Proto-Khoe and Proto-Kalahari Khoe probably emerged further to the north, in an area dominated by !Xun speakers, in line with findings from other linguistic domains (Güldemann, 2004, 2008b, 2019). In line with the archaeological and genetic evidence (Fehn et al., 2022; Lander & Russell, 2018; Oliveira et al., 2023), the modern distribution of Khoe-Kwadi in southern Africa can thus be interpreted as the result of multiple migratory movements from a proto-population along the western fringe of the Kalahari Basin to the north (Kwadi), south (Khoekhoe) and east (Kalahari Khoe), each with its own genetic and linguistic admixture history (Fig. 6).

Taken together, our results indicate that borrowing patterns mirroring the social relations between the Khoe-Kwadi and their Kx’a- and Tuu-speaking neighbours varied across time and space. Early contacts most likely occurred on the western Kalahari Basin fringe and involved significant borrowing of Kx’a and Tuu roots into Proto-Khoe-Kwadi and Proto-Khoe. While vocabulary related to sheep-herding was indeed transferred to Kx’a and Tuu speakers (most notably *g(ʷ)uu ‘sheep’ and *TS’àó ‘to milk’), our data do not suggest a large-scale demic migration akin to the Bantu expansion, which would have entailed the spread of a distinct ethnolinguistic and genetic package from dominant migrants to resident foragers. We note, however, that a more comprehensive analysis of herding-related vocabulary, including practices like corralling, branding and castrating, might provide further insights into contact and migration patterns associated with the spread of livestock – in particular when considered in the context of the archaeological record (see, e.g., Ehret, 2008; Güldemann, 2008b; Vossen, 2017 for a preliminary discussion).

We alternatively suggest that the first Khoe-Kwadi speakers in southern Africa arrived as small-sized groups of hunter-herders whose lifestyle and technological toolkit broadly resembled resident forager communities (cf. Fehn et al., 2022). A significant cultural impact mirrored in unilateral patterns of lexical borrowing from prestigious into less prestigious languages is still attested in areas historically populated by Khoekhoe herders who, in this framework, constitute a later ethnogenetic development, rather than a remnant group retaining the lifestyle of the ancestral migrants (Sadr, 2008; Fauvelle-Aymar & Sadr, 2008).

## 7. Conclusion

Our findings suggest a division between multilateral borrowing within egalitarian forager networks involving speakers of Kx’a, Tuu and Kalahari Khoe from the Central Kalahari, as well as unilateral transfer from dominant Khoekhoe-speaking herders into hunter-gatherer groups along the southern and western Kalahari Basin fringes; Khoe-Kwadi languages participate in both contact settings, in line with the historically observed ethnographic diversity characterizing populations speaking languages of this family.

More generally, we demonstrate how the quantitative analysis of lexical borrowing can provide a rich source of information about the contact history between speakers of different language families, especially in regions for which no written records exist (see also Gerstner-Link, 2023; McMahon et al, 2005). In addition, we show the value of linguistic analyses, together with archaeology and genetics, in tracing migration routes and assessing population contact and admixture.

## Supporting information

10.1017/ehs.2025.10014.sm001 Fehn et al. supplementary material 1 Fehn et al. supplementary material

Fehn et al. supplementary material 2 Fehn et al. supplementary material

## Data Availability

All linguistic data and cognate judgements used in this paper are provided in Supplementary Tables 2–4.

## Supplementary material

The supplementary material for this article can be found at https://doi.org/10.1017/ehs.2025.10014
