# Supplementary material for: Tracing contact and migration in pre-Bantu Southern Africa through lexical borrowing
Source: Evol Hum Sci. 2025 Jul 23;7:e25. doi: 10.1017/ehs.2025.10014 (PMC12516594; doi:10.1017/ehs.2025.10014)
Supplement: Fehn et al. supplementary material 1 — Fehn et al. supplementary material [file S2513843X25100145sup001.pdf]

Supplementary Information for:

Tracing contact and migration in pre-Bantu southern Africa through lexical borrowing

Fehn, Anne-Maria[1,2]*, Sands, Bonny[3], Phiri, Admire[1,2,4], Bolaane, Maitseo[5], Masunga, Gaseitsiwe[6], Fabiano, Ezequiel[1,2,7], Rocha, Jorge[1,2,8]

[1]CIBIO, Centro de Investigação em Biodiversidade e Recursos Genéticos, InBIO Laboratório Associado, Campus de Vairão, Universidade do Porto, 4485-661 Vairão, Portugal.

[2]Biopolis Program in Genomics, Biodiversity and Land Planning, CIBIO, Campus de Vairão, 4485-661 Vairão, Portugal.

[3]Department of English, Northern Arizona University, Flagstaff, AZ, USA.

[4]Department of Linguistics, University of the Free State, Bloemfontein, 9301, South Africa.

[5]San Research Centre, University of Botswana, Private Bag UB 0022, Gaborone, Botswana.

[6]Okavango Research Institute, University of Botswana, Private Bag 285, Maun, Botswana.

[7]University of Namibia, Private Bag 13301, Windhoek, Namibia.

[8]Departamento de Biologia, Faculdade de Ciências, Universidade do Porto, 4099-002 Porto, Portugal.

*Corresponding author: afehn@cibio.up.pt

Supplementary Text S1

1. Historical reconstruction

The cognacy choices and reconstructions presented in this work (Supplementary Table S3) are based on a still-developing corpus of historical-comparative studies focusing on southern African languages. In the following, we will review the studies that our own analysis draws upon.

In contrast to earlier ideas about a "Khoisan" language family encompassing all non-Bantu, non-Cushitic click languages of southern and eastern Africa (e.g. Greenberg, 1963), modern scholars widely accept a classification into three language families in southern Africa (Kx'a, Tuu and Khoe-Kwadi), and two eastern African isolates (Hadza and Sandawe) (Güldemann, 2014a; Sands, 1998). Of the three southern African families, Khoe-Kwadi, has received most attention from historical-comparative linguistics: Vossen et al. (1988) and Vossen (1997) provided a reconstruction of the Khoe branch of Khoe-Kwadi, based on regular sound correspondences and phonological as well as morphological innovations. Vossen's work was later supplemented by Güldemann (2004) and Güldemann and Elderkin (2010) who presented lexical and morphological evidence for a relationship between the Khoe languages and the extinct Angolan language Kwadi, which was eventually consolidated by Fehn and Rocha's (2023) historical-comparative reconstruction of Proto-Khoe-Kwadi. The identification of genuine Khoe-Kwadi roots has also been helped along by the studies of Elderkin on tone (Elderkin, 2008, 2013), vowel reconstruction (Elderkin, 2016) and nasal click accompaniments (Elderkin, 2020), as well as by various studies on the regular loss and replacement of click consonants (Fehn, 2018, 2020a; Traill & Vossen, 1997).

Kx'a as a genealogical unit joining !Xun and ǂ'Amkoe was established by Heine and Honken (2010) and replicated in part by Starostin (2018). Further data on lexical and phonological correspondences within the !Xun subgroup was published by Snyman (1980, 1997), Sands (2010), Heine and König (2015) and Fehn (2020b).

While the Tuu family is widely recognized as a valid genealogical unit, the evidence for this classification is mostly morphological, due to the limited amount of reliable data for the family's !Ui branch (Bleek, 1927; Güldemann, 2005, 2014b; see also Traill, 1975 and Naumann, 2014 for the Taa branch). Nevertheless, lexical roots shared across the family were noted by Bleek (1929), Hastings (2001), Güldemann (2005) and Starostin (2021, 2022). By taking into account historical data from a variety of now extinct !Ui languages (Supplementary Table S1), our work expands on the cognate data available for this severely underdocumented language family.

2. Identification of borrowings and the direction of borrowing

Among the many typological features found in unrelated languages of the Kalahari Basin linguistic area (Güldemann, 1998; Güldemann & Fehn, 2017), the shared phonotactic profile of the Kx'a, Tuu and Khoe-Kwadi families is the most conspicuous. All languages of the area have a fixed set of root patterns (CVV, CVCV and CVN), restrict click consonants to C1, and accept only a small set of consonants in C2 (typically, /b, d, m, n/) (cf., e.g., Güldemann, 2016; Güldemann & Nakagawa, 2018; Nakagawa et al., 2023; Traill, 1980). Because of the shared phonotactic profile, borrowings across SAK families cannot easily be spotted by eyeballing the data and looking for deviant-looking lexical forms (as may be done in more typical contact scenarios that involve phonotactically divergent languages).

To determine the direction of borrowing, we here draw on previously established criteria (cf., e.g., Güldemann & Loughnane, 2012; Haspelmath, 2009), as well as on newly defined criteria taking into account both linguistic ("primary") and extralinguistic ("secondary") features. The full set of discriminatory features considered in this study is outlined and exemplified below.

<u>Primary</u>

A.1 **reconstructability** (regular sound or tone correspondences in one family, but not in another)

Example: The ǂ ~ ǁ ~ ! click correspondence set in Tuu languages (palatal ǂ (Taa-Lower Nossob), lateral ǁ (Nǁng) and alveolar ! (ǀXam and ǁXegwi)) occurs in a fairly large number of roots including basic vocabulary items such as *(g)ʞʰã- 'breast'. The presence of this regular, repeated correspondence is lexically specified and probably traces back to a yet-unspecified click or accompaniment type in the proto-language (Starostin, 2021). The same regular correspondence set, however, is not attested in Khoe-Kwadi. We can therefore tell that Nama (ǁ*khâì* 'chest') borrowed from Nǁng, while Gǀui-Gǁana and ǁAni (ǂ*χãĩ* 'chest') borrowed from Taa.

A.2 **attestation** (restricted attestation in one family, but widespread in another)

Example: The root *ŋǂúú 'black' is attested throughout the Khoe branch of Khoe-Kwadi but restricted to two doculects belonging to the southeastern cluster of the !Xun subgroup of Kx'a. This patterning suggests that *ŋǂúú 'black' originated in Khoe and was borrowed into southeastern !Xun.

A.3 **phonological complexity** (languages in one family include a phoneme or phonotactic pattern unattested in another, assuming that it is easier to lose than to build up complexity)

Example 1: The Taa-root *(ˀ)ŋǂa̤(ˤ)- 'to push' is phonologically more complex than its Khoekhoe attestation ǂ*hàà* (Haacke & Eiseb, 2002). While in Tuu, the root appears with various phonation types not attested in Khoekhoe, Khoekhoe merely reproduces the breathiness through delayed aspiration. For reasons of phonological complexity, we here assume that the origin is located within Tuu, rather than in Khoekhoe.

Example 2: The Kx'a-root *ŋ!a(ˤˀ)ro 'chameleon' is shared between Kx'a and Kalahari Khoe. While it appears with an oral rhyme in Kalahari Khoe, it displays a pharyngealized or pharyngealized-glottalized rhyme in !Xun. Although individual Khoe-Kwadi languages like Naro and Gǀui do have pharyngealization, neither pharyngealized nor glottalized vowels can be reconstructed to the proto-language or any of the family's intermediate stages of reconstruction (pKhoe, pKalahari Khoe, pKhoekhoe). We therefore prefer to assume that the root originated in the family with phonologically more complex forms (Kx'a) and was adapted to the less complex phonology of Kalahari Khoe in the borrowing process.

A.4 **morphological analyzability** (a word is morphologically analyzable in one language or family, but not in another)

Example: The Khoe-Kwadi root *tsoo 'medicine' appears in a compound with Khoe *ǀˀũã 'bone' in parts of Tuu. While this compound originated in Khoekhoe where it is analyzable as 'medicine-bone', it is not analyzable in Tuu and therefore can be assumed to be a borrowing.

A.5 **alternatives** (lexical doublets exist in one family, but not in another)

Example 1: The Nǀuu doculect recorded by Sands and Jones (2022) has two generic words for 'snake', ǀ*χ'əo* and ǀ*ããˤsi*. ǀ*χ'əo* was borrowed from Khoe (Proto-Khoe *ǀx'ao), while ǀ*ããˤsi* is the inherited Nǀuu root also attested in the Nǀuu doculect recorded by Bleek, as well as in the closely related language ǁX'au recorded by Meinhof (1928-9).

Example 2: Doublets do not need to exist within the same doculect, but can also be found within the same cluster of related languages: while *ǂχua 'elephant' is attested throughout both Khoe and Tuu, alternative roots are only found in the Tuu family. For example, Taa West has ǂ*hàbè* (Nakagawa et al., 2023), and the !Ui variety ǁX'au has ǀ*hau* (Meinhof, 1928-9), indicating that Khoe *ǂχua may have replaced inherited roots elsewhere.

Secondary

B.1 **geography** (likelihood of contact by proximity)

Example: It is likely that the !Ui variety ǀXam (Bleek, 1956) borrowed the word for bird, *k'eni*, from Khoekhoe, due to the reconstructability of the root for Proto-Khoe (*kx'ani) and the attested historical contact between the two languages. However, the same argument cannot be invoked for the apparent sharing of a root for 'to dance' between the Northern !Xun doculect recorded by Westphal (1953-1971) (ǀ*xȁ̃*), spoken in southeastern Angola, and the Taa cluster of the Central Kalahari in Botswana and Namibia (*ǀxãã). We therefore consider *k'eni* a borrowing from Khoekhoe into ǀXam, while the sharing of ǀ*xãã* between Northern !Xun and Taa cannot be used to determine the direction of borrowing. Cases like this are frequent in the data and may constitute an echo of a remote common ancestor once shared by Proto-Kx'a and Proto-Tuu, or by ancient contact in an area where languages are not presently neighboring each other.

B.2 **culture** (pastoralism vs. foraging, dominant vs. subordinate)

Example: Roots associated with pastoral culture like *g(ʷ)uu 'sheep' and *TS'ao 'to milk' originated most likely in the Khoe-Kwadi family, which has been associated with the introduction of pastoralism from eastern into southern Africa. In the case of *TS'ao 'to milk', this assumption is reinforced by the presence of an unidentified onset in the proto-language (here represented by the grapheme *TS') which has regular correspondences within Khoe, but irregular correspondences elsewhere, in accordance with different donor languages of the Khoe-branch.

B.3 **historical parsimony** (borrowing into the proto-languages Proto-Kx'a and Proto-Tuu from Khoe-Kwadi is considered unparsimonious, due to the attested late arrival of the Khoe-Kwadi to the Kalahari Basin as compared to the relatively early time period associated with Proto-Kx'a and Proto-Tuu)

Example: As Khoe-Kwadi languages are thought to have arrived in the Kalahari Basin only ~2,000BP, the root *ǀˀa- 'fire' shared between Proto-Tuu and Proto-Khoe-Kwadi is preferentially interpreted as a borrowing from Tuu into Proto-Khoe-Kwadi, rather than the other way around.

To determine the origin of a given root, we commonly used a combination of different criteria. This is illustrated below with four examples combining multiple criteria:

Example 1: The root *dts'V 'to steal' is **attested and reconstructable** (A.1, A.2) across the Kx'a and Khoe-Kwadi families. However, reflexes of the root in Kx'a include prevoicing (Southeastern !Xun) as well as glottalized vowels instead of an ejective onset (ǂ'Amkoe). Both of these **complex phonological features** (A.3) are well attested in Kx'a, but absent in Khoe-Kwadi which neither displays prevoicing nor glottalized vowels. Furthermore, the historical record suggests that Kx'a languages were spoken in the Kalahari Basin before the arrival of the first Khoe-Kwadi speakers. We therefore assume for reasons of **historical parsimony** (B.3) that the root was borrowed from Kx'a into Khoe-Kwadi, and not the other way around.

Example 2: The root *ŋ!!ue 'moon' is attested in !Xun (Kx'a) and Kalahari Khoe (Khoe-Kwadi). The correspondence between a retroflex click in Central !Xun, an alveolar click in southeastern !Xun and a lateral click in Northern and Northwestern !Xun is a **well-attested** sound correspondence in Kx'a and **reconstructs** back to a form with a retroflex click (A.1, A.2). No regular correspondences for retroflex clicks have been established for languages of the Khoe-Kwadi family, and it seems doubtful this click type existed in the respective proto-language (A.3). Therefore, the internal diversity within !Xun is better explained by regular internal correspondences than by borrowing from Kalahari Khoe, which displays a lateral click throughout.

Example 3: The root *ǁʔama ‘to buy’ shows a distribution across all three families, without displaying phonotactic variation. However, closer scrutiny reveals that its distribution in Kx’a and Tuu is **restricted to languages in contact with Khoekhoe** (Central+Southeastern !Xun, Taa West, Lower Nossob, Nǁng) (A.2, B.1). As Khoekhoe is associated with **pastoralism and social dominance** in the area under discussion, it does not seem farfetched to assume that a word for commercial exchange would have originated within the family of the economically dominant party (B.2). Furthermore, a root *(g)ǂṳũ can be reconstructed to proto-Kx’a, making it likely that occurrences of *ǁʔama in Kx’a **replaced the original root** (A.5).

Example 4: The root *ǁχãã ‘to teach’ is attested in languages of all three families. However, it only appears in its reduplicated form and is most likely a fossilized causative. Reduplicated causatives are a Khoe(-Kwadi) feature (Vossen 1997), so the reduplication is **morphologically analyzable** in this family (A.4). Furthermore, its **geographic distribution** outside Khoe-Kwadi overlaps with the influence-sphere of the Khoekhoe herders, namely in Southeastern !Xun, Taa, Nǁng, ǁX’au, and ǀXam (A.2, B.1). It may further be argued that (formal) **teaching is a concept associated with the socially dominant group**, possibly already under the influence of foreign missionaries (B.2).

We refrained from assigning an origin for a root (or for a set of obviously related words) if we were not able to make a conclusive decision, based on the criteria provided above. Two examples for unresolved cases of root sharing across families are provided below:

Example 1: The root *(g)ǁχ’a- ‘to wash’ is widely attested in Kx’a, Tuu, and Khoe-Kwadi. While the presence of prevoicing in Kx’a as well as the presence of different (fossilized?) suffixes in Tuu suggests that the origin is not Khoe-Kwadi, it could not be conclusively decided whether sharing of this root between Kx’a and Tuu is due to areal convergence, or to remote common ancestry.

Example 2: The root *(g)!a̤ˤu ‘cheetah’ is widely attested in Kx’a, Taa and Khoe-Kwadi, but does not display regular correspondences in either unit. While the presence of the complex phonation types pharyngealization, glottality and breathiness in Kx’a and Taa suggests an origin outside Khoe-Kwadi, it was not possible to identify an ultimate donor. We therefore could not decide whether the widespread sharing of this root is due to areal convergence, or to remote common ancestry between Kx’a and Tuu, followed by subsequent reborrowing between individual languages which led to the irregular correspondence patterns observed in the dataset.

Supplementary Figures

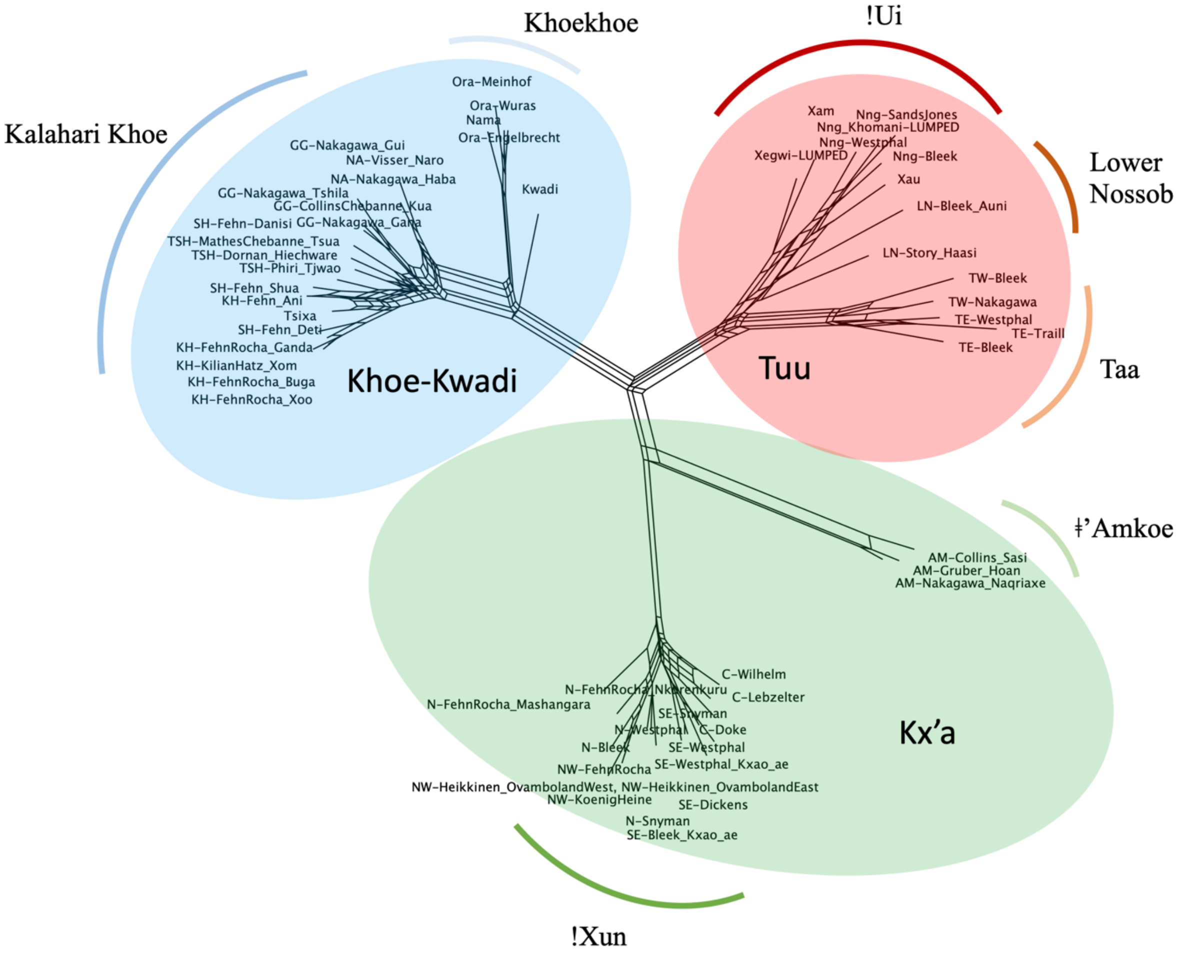

Supplementary Figure S1. NeighborNet analysis of 1,243 cognate sets, excluding singletons, using the same settings as Figure 2A. The Kx'a, Tuu and Khoe-Kwadi families, along with their major subgroups, are clearly identified. Lexical sharing between the three families is evident as reticulation in the center of the network.

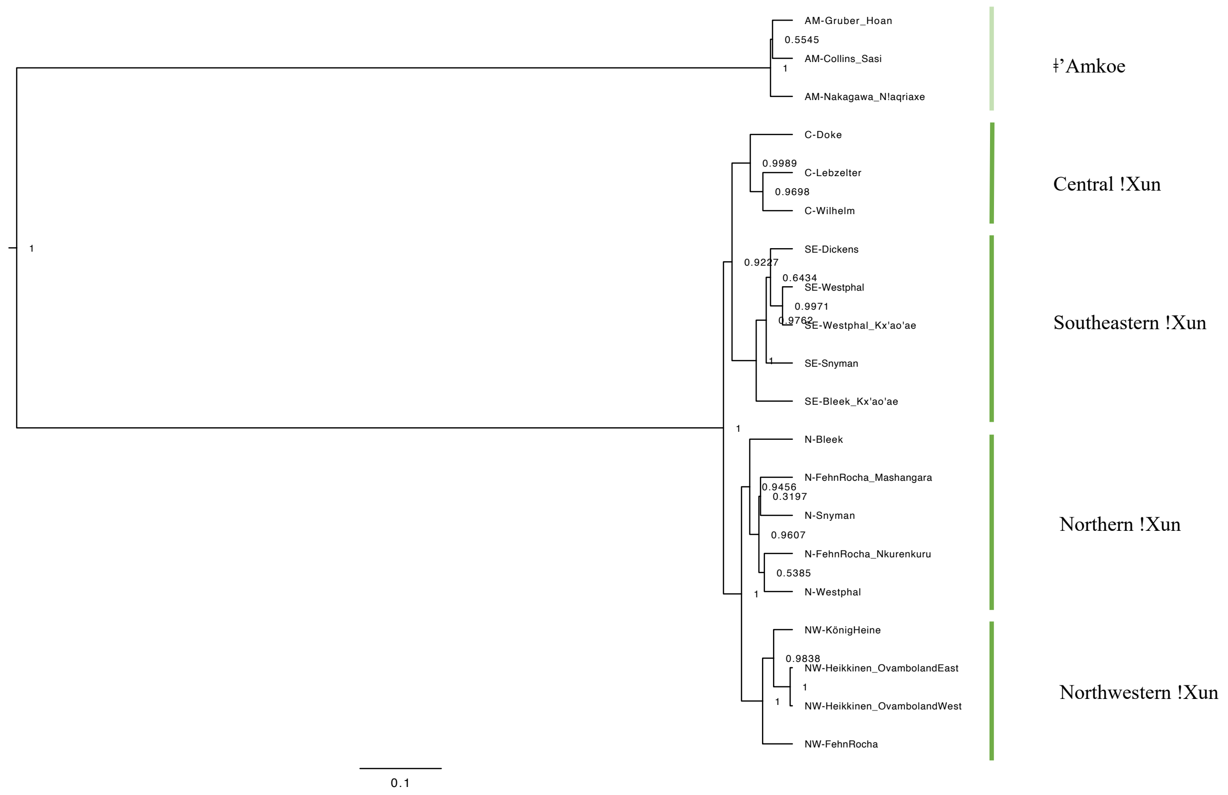

Supplementary Figure S2. Consensus tree of a Bayesian phylogenetic analysis of the Kx'a language family under the Continuous Markov Chain Model, showing a deep split between ǂ'Amkoe (light green) and !Xun (dark green), as well as a relatively shallow differentiation into Central, Southeastern Northern and Northwestern !Xun, in accordance with previous studies (Heine & Honken, 2010; Heine & König, 2015; Sands 2010; Snyman, 1997).

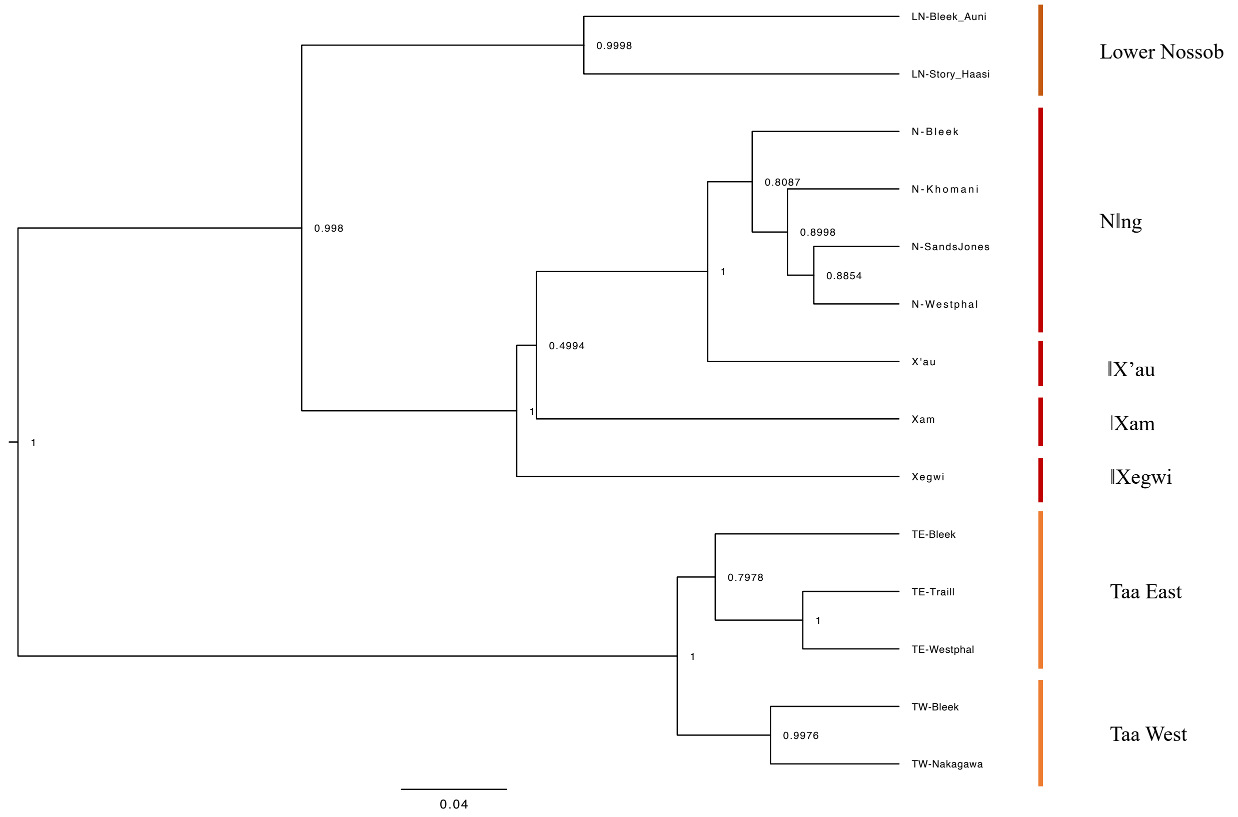

Supplementary Figure S3. Consensus tree of a Bayesian phylogenetic analysis of the Tuu language family under the Continuous Markov Chain Model, delimiting a Taa branch (bright orange) divided into a western and an eastern cluster, and an !Ui branch (red), consisting of the N‖ng cluster including N|uu and ǂKhomani, as well as the isolated doculects ‖X'au, |Xam and ‖Xegwi. The position of Lower Nossob (dark orange) is problematic (see also Starostin, 2022), as grammatical features point towards a closer relationship with Taa than with !Ui (Güldemann, 2014b).

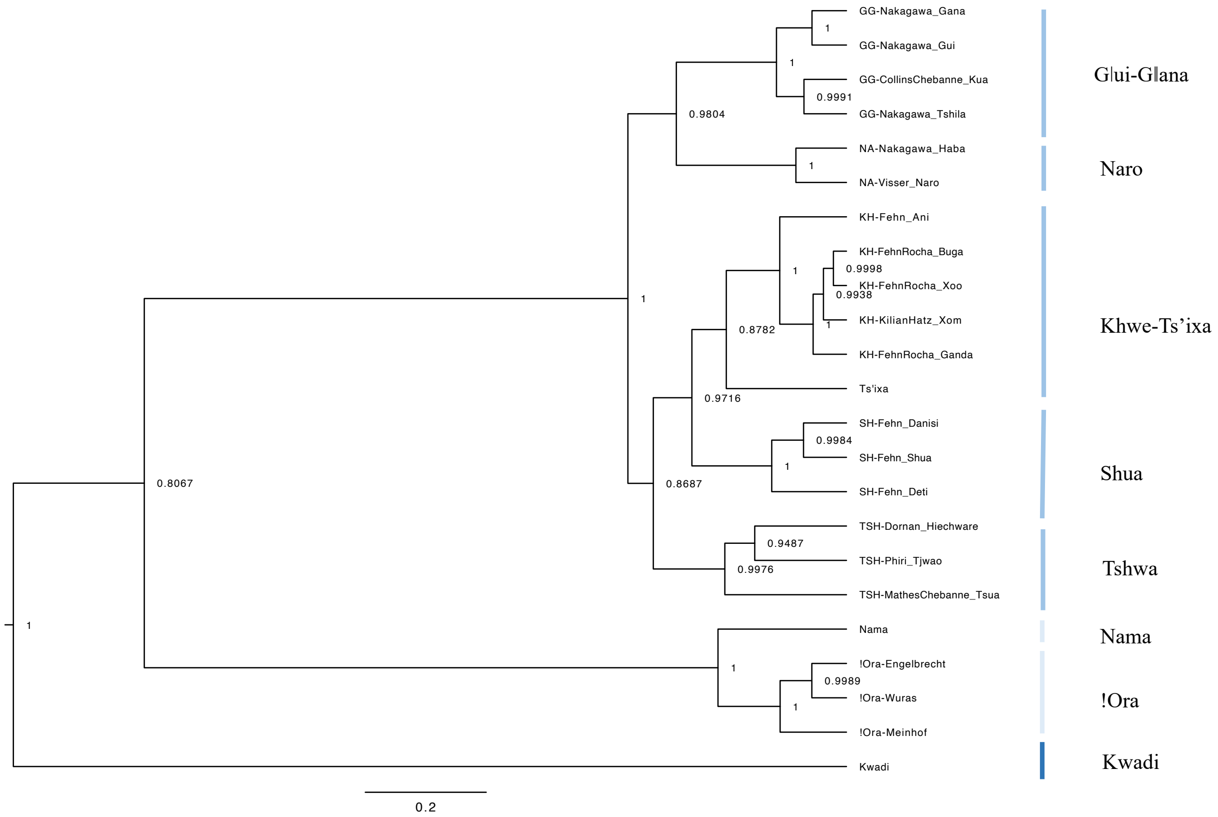

Supplementary Figure S4. Consensus tree of a Bayesian phylogenetic analysis of the Khoe-Kwadi language family under the Continuous Markov Chain Model, showing a major division between Kwadi (dark blue) and the Khoe languages which are further subdivided into Khoekhoe (light blue) and Kalahari Khoe (medium blue) (see also Fehn & Rocha, 2023; Vossen, 1997). Within Khoekhoe, the Nama language can be distinguished from the !Ora cluster represented by three closely related doculects. The Kalahari Khoe subgroup consists of five language clusters: G|ui-G‖ana, Naro, Khwe-Ts'ixa, Shua, and Tshwa.

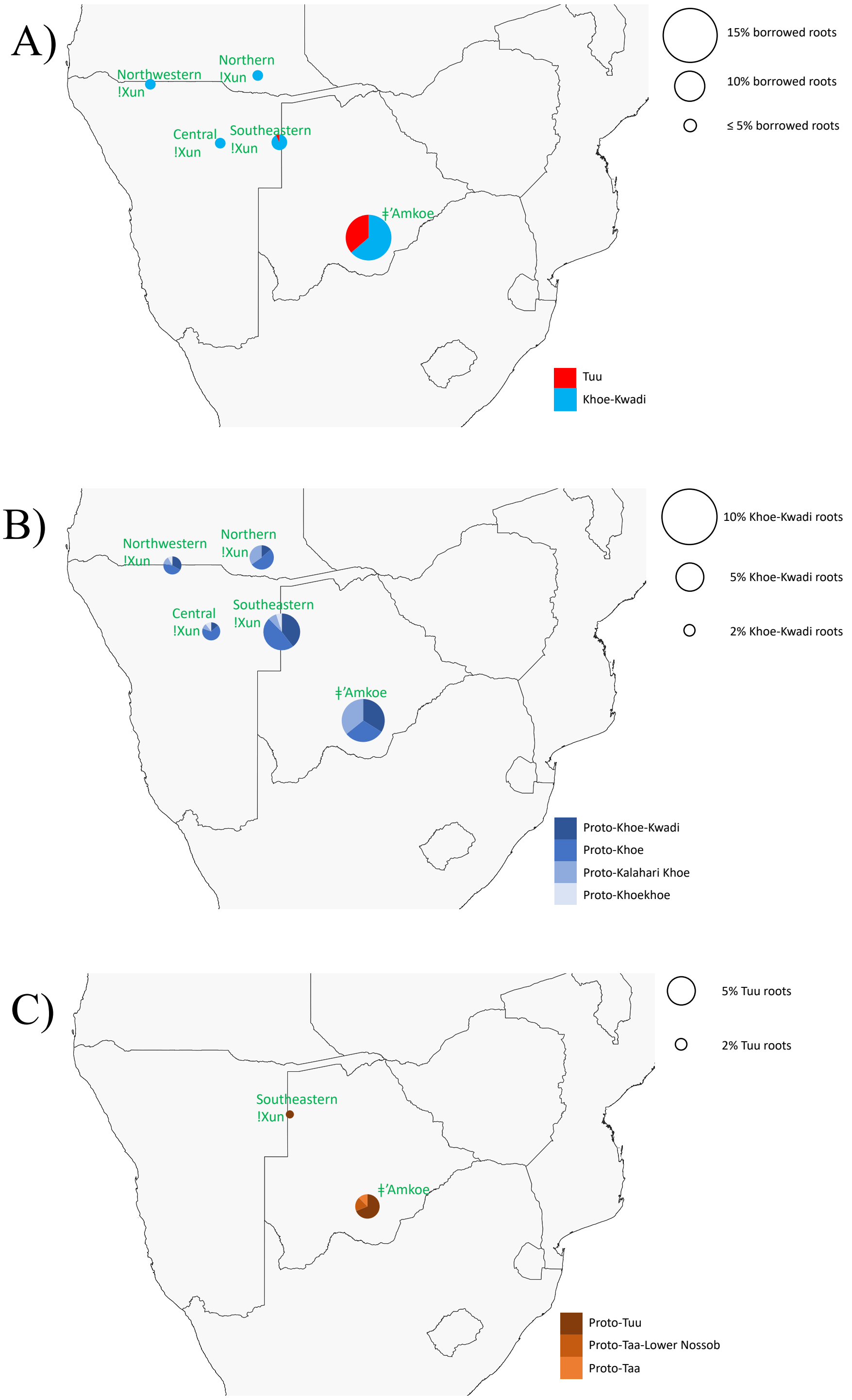

Supplementary Figure S5. Borrowing profiles of languages belonging to the Kx'a family. The size of the pie chart corresponds to the percentage of borrowed vocabulary, as indicated for each figure. A) shows the composition of the borrowed vocabulary in each language; B) shows Khoe-Kwadi borrowings into Kx'a, broken down to the highest resolution level that could be obtained for each root (Proto-Khoe-Kwadi, Proto-Khoe, Proto-Kalahari Khoe, Proto-Khoekhoe); C) shows Tuu borrowings into Kx'a, broken down to the highest resolution level that could be obtained for each root (Proto-Tuu, Proto-Taa-Lower Nossob, Proto-Taa).

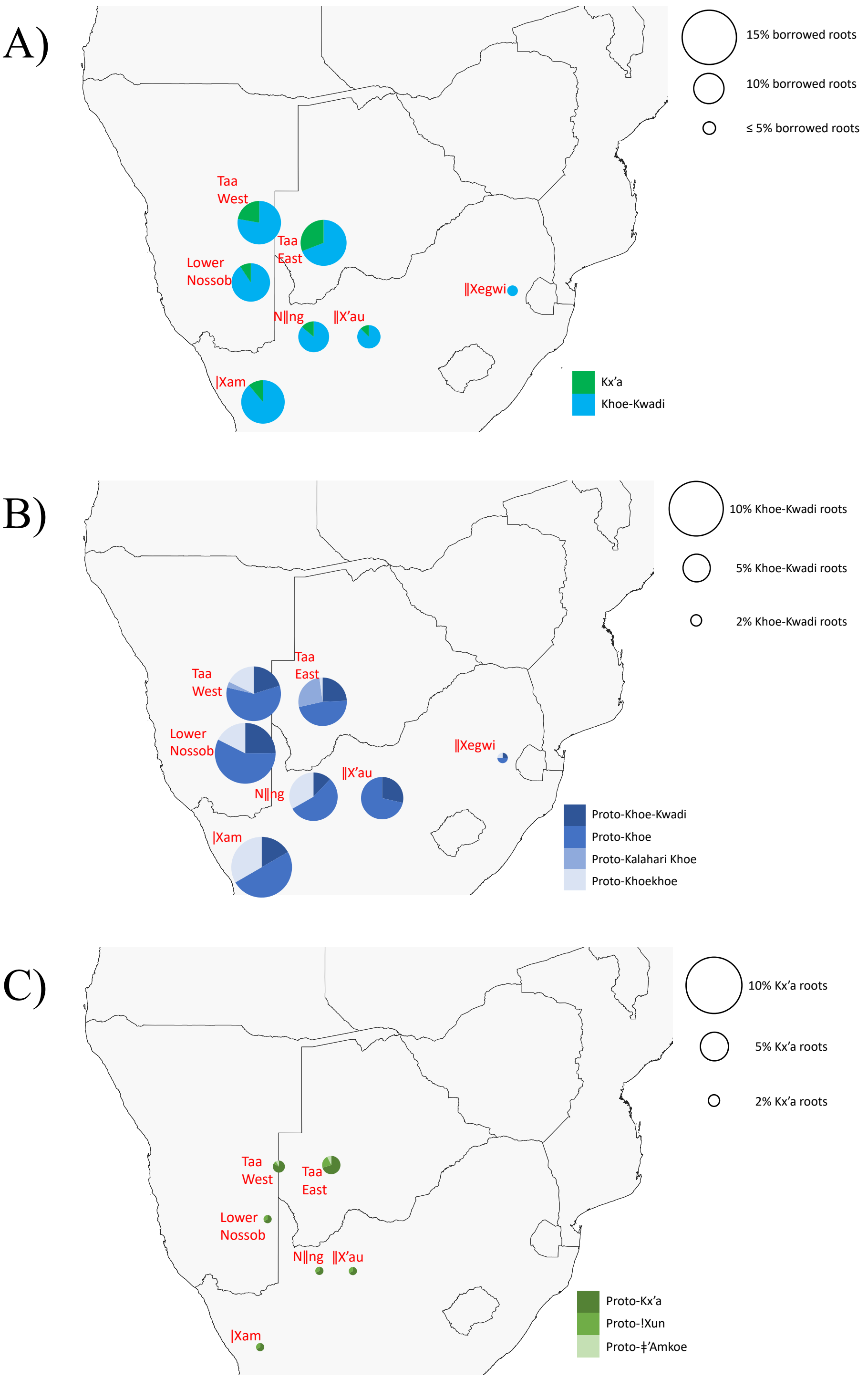

Supplementary Figure S6. Borrowing profiles of languages belonging to the Tuu family. The size of the pie chart corresponds to the percentage of borrowed vocabulary, as indicated for each figure. A) shows the composition of the borrowed vocabulary in each language; B) shows Khoe-Kwadi borrowings into Tuu, broken down to the highest resolution level that could be obtained for each root (Proto-Khoe-Kwadi, Proto-Khoe, Proto-Kalahari Khoe, Proto-Khoekhoe); C) shows Kx’a borrowings into Tuu, broken down to the highest resolution level that could be obtained for each root (Proto-Kx’a, Proto-!Xun, Proto-ǂ’Amkoe).

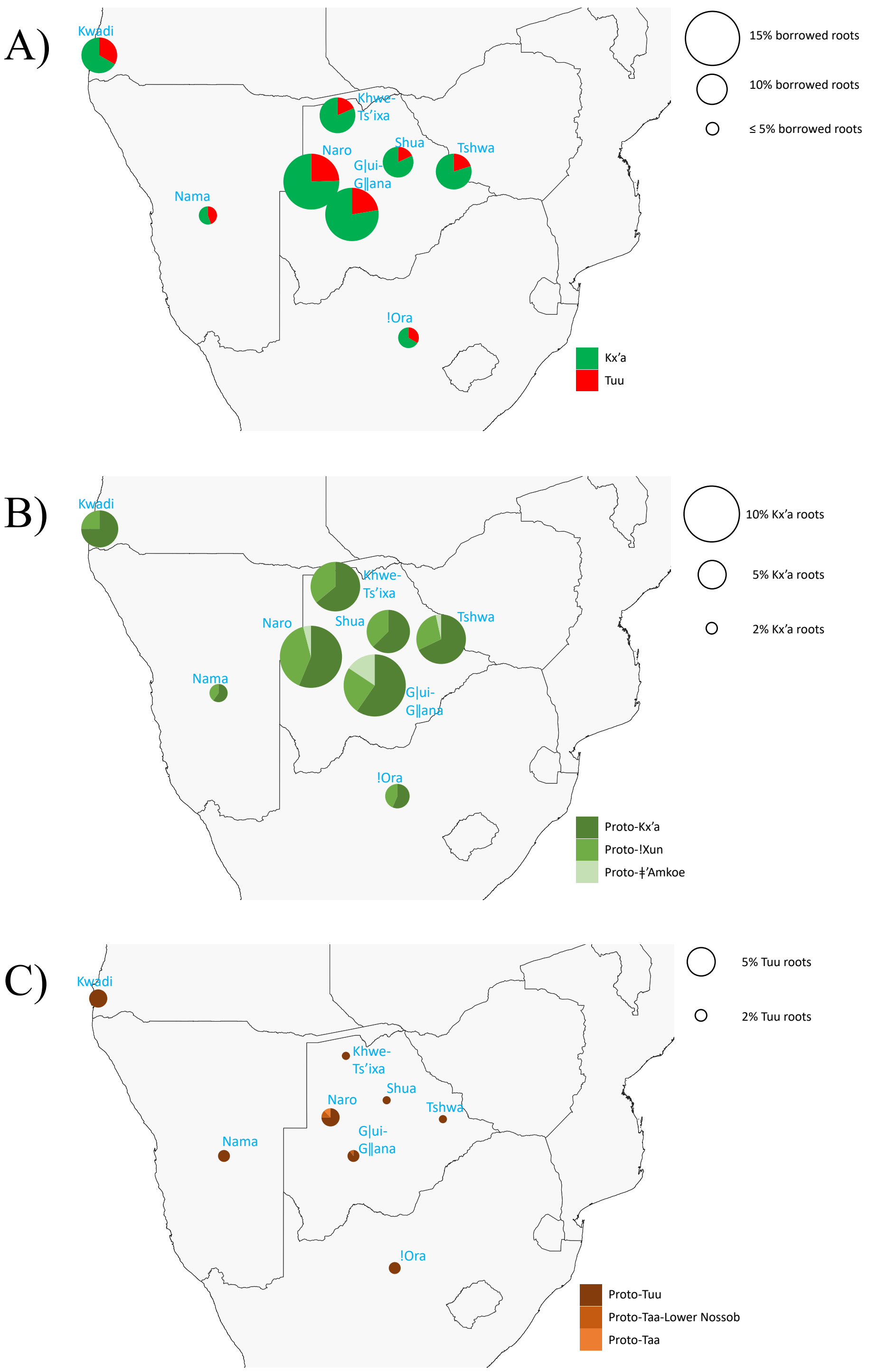

Supplementary Figure S7. Borrowing profiles of languages belonging to the Khoe-Kwadi family. The size of the pie chart corresponds to the percentage of borrowed vocabulary, as indicated for each figure. A) shows the composition of the borrowed vocabulary in each language; B) shows Kx'a borrowings into Khoe-Kwadi, broken down to the highest resolution level that could be obtained for each root (Proto-Kx'a, Proto-!Xun, Proto-ǂ'Amkoe); C) shows Tuu borrowings into Khoe-Kwadi, broken down to the highest resolution level that could be obtained for each root (Proto-Tuu, Proto-Taa-Lower Nossob, Proto-Taa).

References

Bleek, D.F. (1927). The distribution of Bushman languages in South Africa. In F. Boas, O. Dempwolff, G. Panconcelli-Calzia, A. Werner, & D. Westermann (Eds.), *Festschrift Meinhof* (pp. 55-64). L. Friederichsen & JJ Augustin.

Bleek, D.F. (1929). *Comparative vocabularies of Bushman languages.* Cambridge University Press.

Bleek, D.F. (1956). *A Bushman dictionary*. American Oriental Society.

Elderkin, E.D. (2008). Proto-Khoe tones in Western Kalahari. In S. Ermisch (Ed.), *Khoisan Languages and Linguistics: Proceedings of the 2nd International Symposium January 8-12, 2006, Riezlern/Kleinwalsertal* (pp. 87-136). Rüdiger Köppe.

Elderkin, E.D. (2013). Some residual problems of ProtoKhoe lexical tone. In A. Witzlack-Makarevich & M. Ernszt (Eds.), *Khoisan Languages and Linguistics (Proceedings of the 3rd International Symposium July 6-10, 2008, Riezlern/Kleinwalsertal)* (pp. 139-161). Rüdiger Köppe.

Elderkin, E.D. (2016). The vowel system of proto Khoe. In S. Shah & M. Brenzinger (Eds.), *Khoisan Languages and Linguistics: Proceedings of the 5th International Symposium, July 13-17, 2014, Riezlern/Kleinwalsertal* (pp. 53-95). Rüdiger Köppe.

Elderkin, E.D. (2020). The two nasalised accompaniments in proto Khoe. In B. Sands (Ed.), *Click Consonants* (pp. 275-290). Brill.

Fehn, A.M. (2018). New data on Northeastern Kalahari Khoe phoneme inventories: A comparative survey. A*fricana Linguistica, 24*, 5-29.

Fehn, A.M. (2020a). Click loss in Khoe-Kwadi. In B. Sands (Ed.), *Click Consonants* (pp. 291-335). Brill.

Fehn, A.M. (2020b). Click replacement and loss in Ju. In B. Sands (Ed.), *Click Consonants* (pp. 336-255). Brill.

Fehn, A.M. & J. Rocha. (2023). Lost in translation: A historical-comparative reconstruction of Proto-Khoe-Kwadi based on archival data. *Diachronica*, *40*(5), 609-665.

Greenberg, J. H. (1963). *The languages of Africa*. Indiana University.

Güldemann, T. (1998). The Kalahari Basin as an object of areal typology: A first approach. In M. Schladt (Ed.), *Language, identity and conceptualisation among the Khoisan* (pp. 137–169). Rüdiger Köppe.

Güldemann, T. (2004). Reconstruction through 'de-construction': The marking of person, gender, and number in the Khoe family and Kwadi. *Diachronica*, *21*(2), 251–306. https://doi.org/10.1075/dia.21.2.02gul

Güldemann, T. (2005). Tuu as a language family. In T. Güldemann (Ed.), *Studies in Tuu (Southern Khoisan).* University of Leipzig Papers on Africa, Languages and Literatures 23.

Güldemann, T. (2014a). "Khoisan" linguistic classification today. In T. Güldemann & A.-M. Fehn (Eds.), *Beyond "Khoisan": Historical relations in the Kalahari Basin* (pp. 1–41). John Benjamins. https://doi.org/10.1075/cilt.330.01gul

Güldemann, T. (2014b). The Lower Nossob varieties of Tuu. In T. Güldemann & A.-M. Fehn (Eds.), *Beyond 'Khoisan': Historical relations in the Kalahari Basin* (pp. 257–282). John Benjamins. https://doi.org/10.1075/cilt.330.10gul

Güldemann, T. (2016). Phonological regularities of consonant systems in genetic lineages of Khoisan. In R. Vossen & W.H.G. Haacke (Eds.), *Lone Tree: Scholarship in the service of the Koon, Essays in Memory of Anthony T. Traill* (pp. 159-207). Rüdiger Köppe.

Güldemann, T. & Elderkin, E.D. (2010). On external genealogical relationships of the Khoe family. In M. Brenzinger & C. König (Eds.), *Khoisan Languages and Linguistics: Proceedings of the 1st International Symposium, January 4-8, 2003, Riezlern/Kleinwalsertal* (pp. 15-52). Rüdiger Köppe.

Güldemann, T., & Fehn, A.-M. (2017). The Kalahari basin area as a 'Sprachbund' before the Bantu expansion. In R. Hickey (Ed.), *The Cambridge Handbook of Areal Linguistics*. Cambridge University Press. https://doi.org/10.1017/9781107279872.019

Güldemann, T., & Loughnane, R. (2012). Are there "Khoisan" roots in body-part vocabulary? On linguistic inheritance and contact in the Kalahari Basin. *Language Dynamics and Change*, *2*(2), 215–258. https://doi.org/10.1163/22105832-20120205

Güldemann, T. & Nakagawa, H. (2018). Anthony Traill and the holistic approach to Kalahari Basin sound design. *Africana Linguistica*, 24, 45-73.

Haacke, W.H.G. & Eiseb, E. (2002). *A Khoekhoegowab dictionary with an English-Khoekhoegowab index*. Gamsberg Macmillan.

Haspelmath, M. (2009). Lexical borrowing: Concepts and issues. In M. Haspelmath & U. Tadmor (Eds.), *Loanwords in the world's languages: A comparative handbook* (pp. 35–54). Mouton de Gruyter.

Hastings, R. (2001). Evidence for the genetic unity of Southern Khoesan. *Cornell Working Papers in Linguistics 18*, 225–246.

Heine, B., & Honken, H. (2010). The Kx'a family. *Journal of Asian and African Studies*, *79*, 5–36.

Heine, B., & König, C. (2015). *The !Xun language. A dialect grammar of Northern Khoisan*. Rüdiger Köppe.

Meinhof, C. (1928-1929). Versuch einer grammatischen Skizze einer Buschmannsprache. *Zeitschrift für Eingeborenen-Sprachen, XIX*(3), 161-188.

Nakagawa, H., Witzlack-Makarevich, A., Auer, D., Fehn, A.-M., Ammann Gerlach, L., Güldemann, T., Job, S., Lionnet, F., Naumann, C., Ono, H., & Pratchett, L. J. (2023). Towards a phonological typology of the Kalahari Basin Area languages. *Linguistic Typology*, *27*(2), 509–535. https://doi.org/10.1515/lingty-2022-0047

Naumann, C. (2014). Towards a genealogical classification of Taa dialects. In T. Güldemann & A.M. Fehn (Eds.), *Beyond 'Khoisan': Historical Relations in the Kalahari Basin* (pp. 238-301). John Benjamins.

Sands, B. (1998). *Eastern and Southern African Khoisan: Evaluating claims of distant linguistic relationships*. Rüdiger Köppe.

Sands, B. (2010). Juu subgroups based on phonological patterns. In M. Brenzinger & C. König (Eds.), *Khoisan Language and Linguistics: the Riezlern Symposium 2003* (pp. 85–114). Rüdiger Köppe.

Sands, B. & Jones, K. (2022). *N|uuki Namagowab Afrikaans English ǂXoakiǂxanisi/Mîdi di ǂKhanis/Woordeboek/Dictionary.* African Sun Media for African Tongue.

Snyman, J.W. (1980). The relationship between Angolan !Xũ and Žu|'hõasi. In J.W. Snyman (Ed.), *Bushman and Hottentot Linguistic Studies* (Papers of seminar held on 27 July 1979) (pp. 1-58). University of South Africa.

Snyman, J. W. (1997). A preliminary classification of the !Xũũ and Žu|hõasi dialects. In W. Haacke & E. D. Elderkin (Eds.), *Namibian languages: Reports and papers* (pp. 21–106). Rüdiger Köppe.

Starostin, G. (2018). Lexicostatistical studies in Khoisan I: The Ju-ǂHoan relationship. *Journal of Language Relationship*, *16*(1), 19-61.

Starostin, G. (2021). Lexicostatistical studies in Khoisan II/1: How to make a Swadesh wordlist for Proto-Tuu. *Journal of Language Relationship*, *19*(2), 99–135.

Starostin, G. (2022). Lexicostatistical studies in Khoisan II/2: Towards a more precise phylogeny for the Tuu family. *Journal of Language Relationship*, *20*(1), 25–70.

Traill, A. (1975). Phonetic correspondences in the !Xõ dialects: How a Bushman language changes. In A. Traill (Ed.), *Bushman and Hottentot Linguistic Studies* (pp. 77-102). African Studies Institute at the University of Witwatersrand.

Traill, A. (1980). Phonetic diversity in the Khoisan languages. In J.W. Snyman (Ed.), *Bushman and Hottentot Linguistic Studies* (Papers of seminar held on 27 July 1979) (pp. 167-189). University of South Africa.

Traill, A. & Vossen, R. (1997). Sound change in the Khoisan languages: new data on click loss and click replacement. *Journal of African Languages and Linguistics*, 18, 21-56.

Vossen, R. (1997). *Die Khoe-Sprachen: Ein Beitrag zur Erforschung der Sprachgeschichte Afrikas*. Rüdiger Köppe.

Vossen, R., Neumann, S., Patriarchi, C., Rottland, M., Spörl, R. & Vagt, B., (1988). Khoe linguistic relationships reconsidered: The data. In R. Vossen (Ed.), *New perspectives on the study of Khoisan* (pp. 67-108). Helmut Buske.

Westphal, E. O. J. (1953-1971). *The E.O.J. Westphal papers. Manuscript collection.* University of Cape Town.
